# Types of analysis of validation studies in nursing: scoping review*

**DOI:** 10.17533/udea.iee.v40n3e09

**Published:** 2023-02-13

**Authors:** Flávia Barreto Tavares Chiavone, Fernanda Belmiro de Andrade, Anderson Felipe Moura da Silva, Isabelle Campos de Azevedo, Quenia Camille Soares Martins, Viviane Euzebia Pereira Santos

**Affiliations:** 1 Nurse, PhD student. Federal University of Rio Grande do Norte. Rio Grande do Norte, RN, Brazil. Email: flavia_tavares@hotmail.com. Corresponding author. Universidade Federal do Rio Grande do Norte Federal University of Rio Grande do Norte Rio Grande do Norte RN Brazil flavia_tavares@hotmail.com; 2 Nurse, PhD Student. Federal University of Rio Grande do Norte. Rio Grande do Norte, RN, Brazil. Email: fernanda_belmiro_andrade@hotmail.com Universidade Federal do Rio Grande do Norte Federal University of Rio Grande do Norte Rio Grande do Norte RN Brazil fernanda_belmiro_andrade@hotmail.com; 3 Nursing undergraduate student. Federal University of Rio Grande do Norte. Rio Grande do Norte, RN, Brazil. Email: a.felipemour@gmail.com Universidade Federal do Rio Grande do Norte Federal University of Rio Grande do Norte Rio Grande do Norte RN Brazil a.felipemour@gmail.com; 4 Nurse, PhD. Professor. Federal University of Rio Grande do Norte. Rio Grande do Norte, RN, Brazil. Email: isabellebr2511@gmail.com Universidade Federal do Rio Grande do Norte Federal University of Rio Grande do Norte Rio Grande do Norte RN Brazil isabellebr2511@gmail.com; 5 Nurse, PhD. Professor. Federal University of Rio Grande do Norte. Rio Grande do Norte, RN, Brazil. Email: queniacamillesm@gmail.com Universidade Federal do Rio Grande do Norte Federal University of Rio Grande do Norte Rio Grande do Norte RN Brazil queniacamillesm@gmail.com; 6 Nurse, PhD. Professor. Federal University of Rio Grande do Norte. Rio Grande do Norte, RN, Brazil. Email: vivianeepsantos@gmail.com Universidade Federal do Rio Grande do Norte Professor. Federal University of Rio Grande do Norte Rio Grande do Norte RN Brazil vivianeepsantos@gmail.com

**Keywords:** validation study, data analysis, nursing research, nursing., estudio de validación, análisis de datos, investigación en enfermería, enfermería., estudo de validação, análise de dados, pesquisa em enfermagem, enfermagem.

## Abstract

**Objective.:**

To identify and map the types of analysis in nursing validation studies**.**

**Methods.:**

This is a scoping review with collection carried out in July 2020. The following data extraction indicators were considered: year of publication, country of origin, type of study, level of evidence, scientific references for validation and types of analyses. Data were collected in the following bases: U.S. National Library of Medicine, Cumulative Index to Nursing and Allied Health Literature, SCOPUS, COCHRANE, Web of Science, PSYCHINFO, Latin American and Caribbean Literature in Health Sciences, CAPES Theses and Dissertation Portal, Education Resources Information Center, The National Library of Australia's Trobe, Academic Archive Online, DART-Europe E-Theses Portal, Electronic Theses Online Service, Open Access Scientific Repository of Portugal, National ETD Portal, Theses Canada, Theses and dissertations from Latin America.

**Results.:**

The sample consisted of 881 studies, with a predominance of articles (841; 95.5%), with a prevalence of publications in 2019 (152; 17.2%), of Brazilian origin (377; 42.8%), of the methodological study type (352; 39.9%). Polit and Beck stood out as the methodological reference (207; 23.5%) and Cronbach's Alpha (421; 47.8%) as the statistical test. Regarding the type of analysis, the exploratory factor analysis and the content validation index stood out.

**Conclusion.:**

The use of at least one method of analysis was evident in more than half of the studies, which implied the need to carry out several statistical tests in order to evaluate the validation of the instrument used and show its reliability.

## Resumen

**Objetivo.:**

Identificar y mapear los tipos de análisis de los estudios de validación de enfermería.

**Métodos.:**

Esta es una revisión de alcance, con recolección realizada en julio de 2020. Se consideraron los siguientes indicadores para la extracción de datos: año de publicación, país de origen, tipo de estudio, nivel de evidencia, referencias científicas para la validación y tipos de análisis. Los datos se recolectaron en las siguientes bases: U.S. National Library of Medicine, Cumulative Index to Nursing and Allied Health Literature, SCOPUS, COCHRANE, Web of Science, PSYCHINFO, Latin American and Caribbean Literature in Health Sciences, CAPES Theses and Dissertation Portal, Education Resources Information Center, The National Library of Australia's Trobe, Academic Archive Online, DART-Europe E-Theses Portal, Electronic Theses Online Service, Open Access Scientific Repository of Portugal, National ETD Portal, Theses Canada y Tesis y disertaciones de América Latina.

**Resultados.:**

la muestra la constituyeron 881 estudios, con predominio de artículos (841; 95.5%), con una frecuencia mayor de publicaciones en 2019 (152; 17.2%), de origen brasileño (377; 42.8%), del tipo de estudio metodológico. (352; 39.9%). Polit y Beck se destacaron como referencia metodológica (207; 23.5%) y el Alpha de Cronbach (421; 47.8%) como prueba estadística. En cuanto al tipo de análisis, se destacó el análisis factorial exploratorio y el índice de validación de contenido.

**Conclusión.:**

En más de la mitad de los estudios fue evidente la utilización de, al menos, un método de análisis, lo que implicó la necesidad de realizar varias pruebas estadísticas con el fin de evaluar la validación del instrumento empleado y mostrar su fiabilidad.

## Resumo

**Objetivo.:**

Identificar e mapear os tipos de análises dos estudos de validação em enfermagem.

**Métodos.:**

Trata-se de uma *scoping review*, com coleta realizada em julho de 2020. Considerou-se como indicadores de extração de dados: ano de publicação, país de origem, tipo de estudo, nível de evidência, os referenciais científicos para validação e os tipos de análises. Os dados foram coletados nas bases: *U.S. National Library of Medicine*, *Cumulative Index to Nursing and Allied Health Literature*, SCOPUS, COCHRANE, *Web of Science*, PSYCHINFO, Literatura Latino-Americana e do Caribe em Ciências da Saúde, Portal de Teses e Dissertações da CAPES, *Education Resources Information Center*, *The National Library of Australia’s Trobe*, *Academic Archive Online*, *DART-Europe E-Theses Portal*, *Electronic Theses Online Service*, Repositório Científico de Acesso Aberto de Portugal, National ETD Portal, Theses Canada, Teses e dissertações da América latina.

**Resultados.:**

A amostra foi de 881 estudos, com predomínio de artigos (841; 95.5%), com prevalência de publicações em 2019 (152; 17.2%), de origem brasileira (377; 42.8%), do tipo estudo metodológico (352; 39.9%). Sobressaiu-se Polit e Beck como referencial metodológico (207; 23.5%) e Alfa de Cronbach (421; 47.8%) como teste estatístico. Em relação ao tipo de análise destacou-se a análise fatorial exploratória e o índice de validação de conteúdo.

**Conclusão.:**

Em mais da metade dos estudos ficou evidente a utilização de pelo menos um método de análise, o que implicou a necessidade de realização de diversos testes estatísticos para avaliar a validade do instrumento utilizado e mostrar sua confiabilidade.

## Introduction

In the world scenario, there is a growing number of validation studies in the health field aimed at the production of technological resources with the purpose of supporting care in different contexts, both in the training and qualification of professionals, as well as in management and patient care.(1) From this perspective, there are studies aimed at nursing which make it possible to build tools that support the science of this area of knowledge and, consequently, the promotion of a safe, qualified praxis based on scientific evidence.(1,2) It is noteworthy that the validation procedures seek to verify the suitability, quality, legitimacy and credibility of an instrument based on the opinion of experts in the area of the resource theme and/or coming from the view of the target public, to which the validated object is intended.(1,3)

Furthermore, numerous materials can be submitted to validation stages, such as research instruments, care protocols, educational booklets, standard operating procedures, virtual learning objects, algorithms and others.(3) These materials may require one or more types of validation that must be specific and/or adapted, adequate, rigorous and linked to the material elaborated; among the types of validation, the content, cross-cultural, appearance, use and usability validation stand out.(1,4)

Content validation assesses whether the content produced is relevant, correct and adequate for what is proposed.(4) Transcultural validation determines the pertinence of translating an instrument to another reality regarding language and culture.(5) Appearance validation analyses whether the technology built has suitability in relation to graphic and cultural resources and whether it is understandable in terms of the language adopted.(6)

Use validation seeks to trace the degree of user satisfaction with the tool built. As for usability, the validation investigates the effectiveness, efficiency and relevance of the use of the technology in relation to the intended objective in a more broad manner.(6,7) Therefore, each type of validation needs an appropriate analysis of the data, which is based on a rigorous theoretical-methodological framework, since from this analysis it will be determined whether the technology in question is validated and suitable for use or requires adjustments so that it can be effectively used in practice.(1,3)

Thus, the data analysis stage is important for validation studies, as it is considered essential to establish the relevance and quality of the constructed material. However, the reliability of the analysis process must be in line with the material and type of validation performed.(3) Thus, it is essential to investigate the types of data analysis elucidated in the validation studies available in the scientific literature in order to identify and compile the various analysis methods and verify their association with the validation process used. Thus, there is the following guiding question “What types of analysis are used in validation studies in nursing?” and the objective was to identify and map the types of analysis of validation studies in nursing.

## Methods

Study design. This is a Scoping Review with a research protocol registered in the Open Science Framework (DOI:10.17605/OSF.IO/YH9UZ) based on the recommendations of the Joanna Briggs Institute (JBI) Reviewer's Manual,(8) according to the proposed stages in the Preferred Reporting Items for Systematic reviews and Meta-Analyses extension for Scoping Reviews (PRISMA-ScR): Checklist and Explanation(9) according to the theoretical framework of Arksey and O'Malley.(10) This type of study seeks to identify and map the main concepts that support an area of knowledge through the survey of scientific productions and relevant studies in order to list the existing gaps in the literature. For this, the following stages are adopted: 1) delimitation of the research question; 2) identification of relevant studies; 3) selection of studies; 4) data mapping and; 5) grouping, summarization and presentation of the results.(10) With regard to the elaboration of the research question and descriptors listed, the Population, Concept and Context (PCC) strategy was used with the use of descriptors in health sciences (DECS ) and their correspondents in English indexed in the Medical Subject Headings (MESH), where P (population) - validation study, C (concept) - data analysis and C (context) - nursing research. Thus, the following question was established: What types of analysis are used in validation studies in nursing?

Study location and data collection period. A primary investigation was carried out by crossing Data analysis AND Validation study AND nurse research in the National Library of Medicine (PUBMED) and Cumulative Index to Nursing and Allied Health Literature (CINAHL) databases to identify and survey the most common keywords frequently in surveys. Thus, the search strategy was obtained with the help of the Boolean operators AND and OR: Validation Study OR (Validation OR Instrument Validation) AND Data Analysis OR (Analysis) AND Nurse Research OR (Nursing Sciences). It should be noted that the search in each database was adapted to its specific search engines, but the compatibility of descriptor combinations was maintained. The capture of studies was performed in July 2020. The following platforms were used to search for articles: U.S. National Library of Medicine (PUBMED), Cumulative Index to Nursing and Allied Health Literature (CINAHL), SCOPUS, COCHRANE, Web of Science, PSYCHINFO, Latin American and Caribbean Literature in Health Sciences (LILACS). In view of the theses and dissertations that make up the gray literature, the following were explored: CAPES Thesis and Dissertation Portal, Education Resources Information Center (ERIC), The National Library of Australia's Trobe (Trove), Academic Archive Online (DIVA), DART- Europe E-Theses Portal, Electronic Theses Online Service (EThOS), Open Access Scientific Repository of Portugal (RCAAP), National ETD Portal, Theses Canada, Theses and dissertations from Latin America.

Selection criteria. The following inclusion criteria were defined for the eligibility of the studies: research related to the types of analysis of validation studies in nursing, studies published in full and available electronically on the CAPES Periodicals Portal through the Federated Academic Community (CAFe). No restrictions were established regarding language and time of publication. Editorials, letters to the editor, opinion articles and reflection articles were excluded. In addition, duplicate materials were counted only once.

Sample definition and Study variables. The study selection process took place in two stages, the first starting from reading the titles and abstracts, which were verified by peer reviewers. In the second stage, the studies were analyzed by reading the material in full and the indicators were extracted: year of publication, country of origin, type of study, level of evidence based on the JBI recommendations,(11) which consists of the framework that guides the scoping review, the scientific frameworks for validation and the types of analysis (tests, statistical calculations, etc.)

Data processing and analysis. The selected data were organized in a spreadsheet using the Microsoft Excel 2010® software, evaluated using simple descriptive statistics, and presented in graphs, tables and figures.

## Results

**Figure 1:**
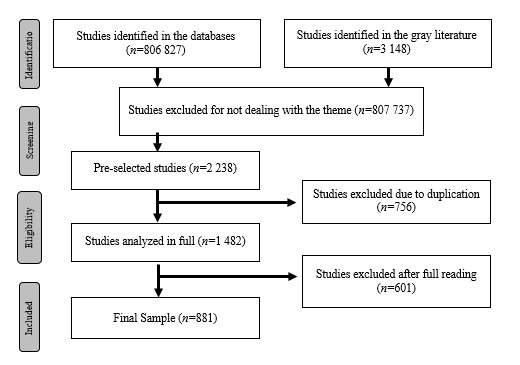
Flowchart of the study search and selection process

From the search in the databases, 809,975 studies were initially identified, which were analyzed in stages that are described in Figure 1, so that a final sample of 881 studies was obtained.

**Figure 2:**
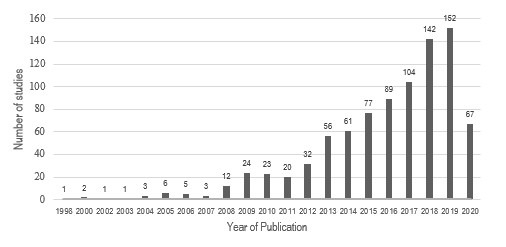
Distribution of years in which the 881 studies that made up the sample were published

The selected studies consist of articles (841; 95.5%), dissertations (21; 2.3%) and theses (19; 2.2%). With regard to the period of publications, the year 2019 stood out (152; 17.2%) (Figure 2).

**Figure 3:**
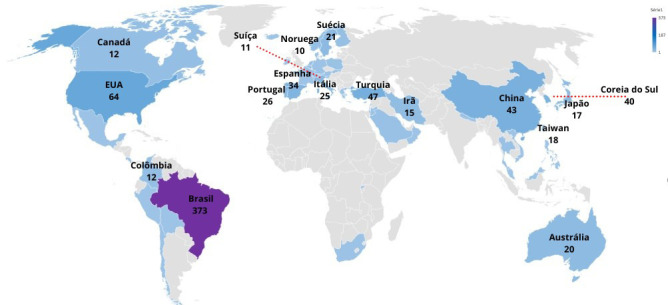
Countries where the studies were developed

Regarding the origin of the studies, Brazil (377; 42.8%), the United States of America (64; 7.2%) and Turkey (47; 5.3%) stood out, as shown in Figure 3.

Regarding the type of method, there was a prevalence of those classified as methodological (352; 39.9%), cross-sectional (163; 18.5%), validation studies (125; 14.2%), descriptive (50; 5.6%), observational (14; 1.5%), cohort and exploratory (6; 0.7%), quasi-experimental (4; 0.4%), accuracy, concept analysis, randomized clinical trial and action research (2; 0.2%) each, case control, sequential triangulation and systematic review (1; 0.1%) each. It is noteworthy that some studies (149; 16.9%) did not describe the method used. The levels of evidence of the studies were evaluated, in which there was a predominance of level five (477; 54.1%), followed by level four (238; 27.0%), level three (9; 1.0%), level two (4; 0.5%), and level one (3; 0.3%). In addition, the data collection indicators (main methodological references applied in the analysis of validation studies in nursing and the tests and statistical calculations performed) were synthesized and presented in Table 1.

**Table 1:** Synthesis with the references and methodological references used in the analyses of the 881 validation studies in nursing and the tests and statistical calculations performed

Indicators of the collection	**Main Findings *n* (%)**
References cited for the validation analyses	Polit and Beck - 207 (23.5%)
	Hair - 44 (5.0%)
	Tabachnick and Fidell - 38 (4.3%)
	Pasquali - 30 (3.4%)
	Nunnaly and Bernstein - 30 (3.4%)
	Hu and Bentler - 26 (2.9%)
	Lynn - 24 (2.7%)
	Fehring - 21 (2.4%)
	Streiner and Norman - 19 (2.2%)
	Kline - 18 (2.0%)
	Cohen and Waltz - 17 (1.9%)
	DeVellis - 13 (1.4%)
	Altman - 10 (1.1%)
	Figueiredo-Filho - 5 (0.6%)
	Linacre - 4 (0.5%)
	Fabrigar - 3 (0.3%)
	Sampieri, Collado and Lucio - 3 (0.3%)
	Bandura - 2 (0.2%)
	Muthen and Muther - 2 (0.2%)
	Salkind - 1 (0.1%)
	Not appointed - 182 (20.7%)
Statistical tests and calculations	Cronbach's Alpha - 421 (47.8%)
	Kaiser-Meyer-Olkin test - 254 (28.8%)
	Bartlett's sphericity test - 224 (25.4%)
	Descriptive statistics - 86 (9.8%)
	Student t test - 54 (6.1%)
	Chi-square test - 38 (4.3%)
	Pearson - 34 (3.9%)
	ANOVA test - 33 (3.7%)
	Mann-Whitney Test - 25 (2.8%)
	Kolmogorov-Smirnov test - 18 (2.0%)
	Binomial Test - 13 (1.5%)
	Kruskal-Wallis test - 13 (1.5%)
	Shapiro-Wilk Test - 8 (0.9%)
	Wilcoxon Test - 8 (0.9%)
	Guttman Test - 7 (0.8%)
	Arl Test - 6 (0.7%)
	Friedman Test - 4 (0.5%)
	Kuder-Richardson Thesis - 4 (0.5%)
	Sam's Test - 4 (0.5%)
	Youden Test - 4 (0.5%)
	Horn Parallel Analysis - 3 (0.3%)
	Lawshe Method - 3 (0.3%)
	McDonald's Omega - 3 (0.3%)
	Cluster Analysis - 2 (0.2%)
	Aiken's V coefficient, - 2 (0.2%)
	Iter-item coefficient -2 (0.2%)
	Inter-total correlation - 2 (0.2%)
	Lin concurrency coefficient -2 (0.2%)
	Duncan's Test - 2 (0.2%)
	Turkey-Kramer Test - 2 (0.2%)
	Calinski-Harabasz Index - 1 (0.1%)
	Dunn's Test - 1 (0.1%)
	Hotelling T^2^ Test - 1 (0.1%)
	Levene Test - 1 (0.1%)
	Cattell's Scree Test - 1 (01%)
	Mean variance extracted - 1 (01%)

There were also variables such as the types of analysis of validation studies in nursing and the amount of analysis used in each research, which are described in Table 2.

**Table 2:** Synthesis of the types of analysis of the 881 validation studies, with the collection indicators: types of analysis and amount of analysis used

Indicators of the collection	Main Findings
Types of analysis	Exploratory factor analysis - 360 (40.9%)
	Content validity index - 346 (39.3%)
	Confirmatory factor analysis - 213 (24.2%)
	Intraclass correlation coefficient - 166 (18.8%)
	Pearson's correlation coefficient - 134 (15.2%)
	Agreement among judges - 85 (9.6%)
	Kappa agreement coefficient - 80 (9.1%)
	Spearman rank correlation- 68 (7.7%)
	Accuracy measures - 15 (1.7%)
	Kendall tau correlation coefficient - 10 (1.1%)
	Rasch model. Content validity ratio - 8 (0.9%)
	Diagnostic content validity - 7 (0.8%)
	C-Score. Weight - 5 (0.6%)
	Multi-trace-multi-method analysis - 3 (0.3%)
Number of analyses used in the studies	One type of analysis - 440 (50%)
	Two types of analysis - 266 (30.2%)
	Three types or more - 176 (19.8%)

## Discussion

Regarding the analysis of published studies, the predominance of articles was noticed. This finding is associated with the fact that this type of production is carried out in a shorter period of time compared to the others and, often, they consist of clippings from larger research, such as research reports, dissertations and theses.(12) Regarding the year of publication, 2019 stood out, which is in line with the growing popularization of validation studies to produce and legitimize quality instruments, which are essential to support and structure nursing care, with a view to reinforcing it as a science, as it proposes a practice based on literature and scientific knowledge.(13) It is noteworthy that such progression in the construction of these studies over the periods may have also been driven by the launch of the “Nursing Now” campaign, which aims to promote the appreciation of the profession by the end of 2020 through investment in the production of knowledge, technical-scientific advancement and health education, thus encouraging the development, the innovation of nursing science and the production of technologies in the area.(14)

When analyzing the origin of the materials that made up the sample, a significant portion of Brazilian archives was evidenced, which is supported by the gradual expansion of *stricto sensu* graduate courses in the health area, which foster the progress of science and technology, which require a careful evaluation before their implementation and, therefore, validation studies become essential as they attest to the quality of the tool developed.(15) Regarding the type of method used in the analyzed studies, there was a prevalence of methodological studies to the detriment of the others, which can be understood by its delimitation, since it allows the researcher to build his validation project structured in stages, from the idealization, search in the literature, elaboration and proof of its reliability for practice, therefore, it is more employable and, thus, more accepted.(16)

The data related to the references and benchmarks used in the validation analysis show Polit and Beck as the ones that obtained the highest number of citation. This finding can be explained by the wide dissemination of their works and the international recognition of the authors identified as specialists in research methods in health and nursing.(16) Another highlighted reference was that of Pasquali, who directs his assumptions to psychology, but is constantly adapted by health researchers to the area, generally associated with the need for validation of criteria, content and construct.(1) Pasquali's psychometry is marked by the completeness of its process, as it provides support for the construction and validation of the instrument, thus contemplating the necessary stages in a clear and objective way for the material to be considered usable, such attributes make the reference preterable among the others.(1,17)

With regard to tests and statistical calculations, the prevalence of Cronbach's Alpha was verified. This fact corroborates a study(18) carried out in 2017, which states that this is the most suitable for evaluating the estimate of the internal consistency of an instrument and is based on the degree of covariance between the items. Therefore, with Cronbach's alpha, the researcher is able to determine if the instrument developed is in line with the target audience, if it is repetitive and if it is consistent for what it intends to measure.(19) Another highlighted statistical calculation was the Kaiser-Meyer-Olkin (KMO) Test, which refers to whether the Factor Analysis (FA) is appropriate to the research data, and this is directly associated with the Bartlett's Sphericity Test, which presents the adequacy of the FA according to the hypothesis testing of the correlation and identity matrix.(20)

Thus, research(21) highlights the importance of the KMO test by allowing the verification and adjustment of variables as a fundamental process in validation studies, as they directly influence the constitution of the factors that represent the construct. Regarding the types of analysis, there was a predominance of factor analysis, which is classified as exploratory and confirmatory. Exploratory factor analysis in a validation procedure allows attesting to the representativeness of the data, thus making the material developed more succinct and objective, by grouping similar items.(22) While confirmatory, as the name implies, confirms the structure and construct of the instrument, considered indispensable in the validation processes, as it directs the management of the necessary factors so that the material does not deviate from its proposal.(18)

Still on the types of analysis, the wide use of the Content Validity Index (CVI) was found as one of the most important dimensions in the analysis of a material, since it allows the determination of the validation of the material based on statistical calculations defined according to the pre-established.(18,23,24) The CVI is in line with Polit and Beck, who guide the performance of this calculation in three ways: through the mean of the proportions of the items classified as relevant, through the sum of each CVI individual divided by the total number of items, or the sum of all relevant items divided by the total number of items.(19)

However, for Pasquali, this process is understood as Content Validation Coefficient (CVC) and differs in statistical calculations. In this case, each item and the instrument as a whole are validated according to previously established criteria and by means of an expert opinion, which provides the adequacy of the content and semantics of the material to make it understandable to the audience for which it is intended and compatible with the purpose for which it is available.(25) Both verify the suitability, representativeness and relevance of the instrument in relation to the phenomenon that it aims to measure. It is highlighted as a relevant characteristic to the subjectivity of these methods when considering the individuality in the interpretation of each judge and, consequently, can result in distortions in the evaluation.(1,26)

It is evident that the presence of one more method of analysis in 50% of the studies implies the need for several tests to measure the validation of a material and prove its reliability. These results corroborate an integrative review(27) by confirming the use of multi-methods in data analysis. Finally, the data extraction procedures are highlighted as a limitation of the study, since many studies that composed the sample described the data analysis stage succinctly and some did not fully portray the types of analysis and statistical calculations used to validate the material produced, which may imply limitations of the findings presented.

## Conclusion

The main types of analysis used in validation studies in nursing are the exploratory factorial and the content validation index. Regarding the methodological framework, Polit and Beck stood out, and as a statistical test, Cronbach's Alpha for structuring and evaluating the tool produced. All these processes allow the improvement of the technologies developed and ensure their quality, in addition to enabling their use in health services, since such materials guide and the routine of professionals and turn it dynamic by subsidizing their practical performance and strengthening the theoretical basis.

Furthermore, as contributions from the research developed, the mapping of the analyzed information is elucidated, considering that from the results obtained, researchers will be able to base the analyses of their validation studies by understanding which types of analyses are most suitable for each type of approach according to the findings of this material, as well as statistical tests and calculations that can be associated and appropriate references and benchmarks
